# Supplementary material for: Association between fall-related serious injury and activity during fall in an acute care hospital
Source: PLoS One. 2023 Jul 7;18(7):e0288320. doi: 10.1371/journal.pone.0288320 (PMC10328302; doi:10.1371/journal.pone.0288320)
Supplement: Attachment — Submitted filename: Response to Reviewers #2.docx [file pone.0288320.s004.docx]

**Dear Reviewer #2**

Thank you very much for providing important comments. We are thankful for the time and energy you expended. We are delighted to hear that you think our work will spark debate in our field. In the following sections, you will find our responses to each of your points and suggestions. We are grateful for the time and energy you expended on our behalf.

1. ***Page 8, row 180-181: The authors described that "serious injury resulting from a fall occurred when the patient was in an unassisted situation". However, I didn’t find a significant difference between them in Table 1. Therefore, based on the current study results, the sentence was inappropriate.***

- We agree with your assessment. We have totally deleted the sentence.

2. **Page 8, row 192-193: Please provide any evidence to support the sentence "Patients in an acute care hospital have an acute change in their body and mental functional status and environment", the logic that the change causes falls while walking, and the sentence "Therefore, to prevent injuries caused by falls, it is important to evaluate patients’ ability to walk at several appropriate times".**

- Thank you for your suggestion. We have reflected this suggestion as Page 10, row203-209. We hope that you agree.

3. ***Page 9, row 197-204: This paragraph mentioned the possibility for difference between fall rates of inpatients with injuries in previous studies and the current study. However, the rates were similar with each other and the rates were not statistically judged, and the subjects of those studies were different, which were not considered in the logic. The discussion in this paragraph lacked evidence.***

- We agree with your assessment. We have totally deleted the sentence.

4. **Page 9, row 211-213: The authors said that "Our results showed that the group of patients with serious injuries were less likely to have a history of falls". However, the p-value was 0.462 in Table 1, which was not low even relatively. It could not be said that they were "less likely".**

- We agree with your suggestion. We have reflected this suggestion as Page 10-11, row221-225. We hope that you agree.

5. **Page 10, row 238-239: The sentence "As previously reported in a study of injured athletes, video analysis can provide insights into the mechanisms of injury and can help to devise preventative strategies [34]." seemed not to be relevant to the current study results. I suggest to delete this sentence.**

- We have totally deleted the sentence.

6. **Page 11, row 249-251: The statement "Because the patient's condition is easily changed in an acute care hospital, it is important to evaluate the patients’ ability to walk at several appropriate times to prevent injury caused by falls." had the problem as the same as ②, which had a logical leap. Please revise appropriately.**

- We have reflected this suggestion as Page 12, row261-267. We hope that you agree.

Again, we appreciate all of your insightful comments. We worked hard to be responsive to them. Thank you for taking the time and energy to help us improve the paper.

Best regards,

Kosuke Kobayashi, Ph.D, PT
